# Global Adversities, the Media, and Mental Health

**DOI:** 10.3389/fpsyt.2021.809239

**Published:** 2022-01-10

**Authors:** Ladislav Kesner, Jiří Horáček

**Affiliations:** ^1^National Institute of Mental Health, Klecany, Czechia; ^2^Faculty of Arts, Masaryk University, Brno, Czechia; ^3^Third Faculty of Medicine, Charles University, Prague, Czechia

**Keywords:** adversity, uncertainty, media news, stress, inflexibility

## Abstract

Global communities are currently confronted with a number of complex problems and threats, the reality of which is amplified by the media. These environmental and socio-political stressors have been accompanied by the spread of problematic psychological and behavioural tendencies, such as the growing polarisation of opinions and values, online radicalisation and extremism, deepening xenophobia and nationalism, the proliferation of irrational beliefs and conspiracy theories, and resistance to rational public policy measures. Here we argue that although they fall outside the scope of psychopathology, they nevertheless currently constitute a major challenge for psychiatry as a research domain and a clinical practise. To substantiate this claim, we outline the mechanisms by which media-transmitted stressors impact mental well-being and possibly psychopathology. The common denominator of these global problems and the media's construction of reality is the increase in uncertainty, unpredictability, and uncontrollability, which prompts defensive responding and, in predisposed individuals, functions as a potent source of chronic stress. These contribute to cognitive inflexibility, a strong predisposing factor for the development of rigid beliefs and attitudes, which to varying degrees underlie the adverse psychological and behavioural tendencies mentioned above. We suggest that the tightening of beliefs and ideas that is the result of cognitive rigidity may correspond to the clinical characteristics of induced delusional disorder. This can be seen as a (ultimately maladaptive) defensive strategy for coping with a high degree of uncertainty and unpredictability. We conclude by briefly outlining the possible ways in which psychiatry can face this challenge.

Anyone who risks deeper immersion in the news media might be excused for harbouring a pessimistic outlook on the state of the world. Global communities are currently confronted with a plethora of complex problems and threats: advancing and potentially catastrophic climate change, accelerating economic inequality, renewed hostility among superpowers and a mounting arms race, widespread displacement and migration, the resurgence of authoritarian political tendencies in many parts of the world, and the global COVID-19 pandemic—to name only the most obvious ones. Every day, millions of people are affected by upheavals, political conflicts, and natural disasters. These problems have been accompanied and amplified by the spread of problematic psychological and behavioural tendencies, such as a steep increase in the polarisation of opinions and values, radicalisation and extremism, deepening xenophobia and nationalism, the proliferation of irrational beliefs and conspiracy theories, resistance to rational public policy measures, and the viral diffusion of negative emotions in the public space and on social media. People's mental states are being massively manipulated with the use of new media, on a scale unprecedented since WW II. Opinion-makers and scientists have repeatedly drawn links between the complicated state of the world and people's psychological well-being and there are widespread concerns that these phenomena constitute a threat to social health and are undermining democratic principles. Indeed, the humanistic concerns about the collective sanity of society that were raised by some thinkers in the (mid-) twentieth century resonate powerfully in the world today (1).

The above-mentioned adverse behavioural phenomena have been addressed by novel research in social psychology, cognitive science and neuroscience. In contrast to these efforts, psychiatry has so far been rather reticent in responding to them, despite the fact that it must certainly deal with their consequences. One exception is the growing literature on the relationship between mass violence and mental health (2–4) and assesments of acts of extremist violence in forensic psychiatry (5–7). But overall, psychiatry has not yet sufficiently reacted to the spread of negative collective mental states, that are the result of the real or perceived threats confronting global communities and the ways they impact individual mental well-being. Here, we argue that the mental and behavioural problems that are part and parcel of the current complicated state of the world and the individual responses to it, currently constitute a major challenge for psychiatry as a research domain and a clinical practise. To substantiate this claim, we will here briefly outline the mechanisms by which media-transmitted stressors impact mental well-being.

## An Ethical and A Political Challenge

A consistent line of thinking that spans both extreme critical positions and more mainstream views has long insisted on the need to demarcate mental illness from other forms of mental distress that are caused by social problems (8, 9). This position is explicitly embraced in current diagnostic schemes, where suffering and maladaptive behaviour that are the result of social circumstances are distinguished from mental disorders. According to DSM-5, mental suffering, socially deviant behaviour, and conflicts that exists primarily between the individual and society “… are not mental disorders unless the deviance or conflict results from a dysfunction in the individual….” [(10), p.20]. Even the socially most destructive phenomena, such as terrorism and mass violence, presuppose cognitive distortions (11) but cannot simply be accounted for in terms of mental pathology. It could thus be argued that psychiatry's reticence about the above-mentioned negative stereotypes, beliefs, and behaviours is in fact justified: while they are to various degrees morally repulsive and socially destructive—and indeed pathological in social sense—their links to psychopathology are tenuous. But while there is an obvious imperative to resist the psychiatrisation of new and pressing social pathologies, to assert that psychiatry should stay clear of these murky waters is no less problematic—and in our view untenable—for at least two principal reasons.

The first reason becomes apparent if we accept that the social mandate of psychiatry is not just the treatment of mental disorders but includes also the preservation of mental health. Under the well-established dual-factor or two-continua models of mental health and illness (12) and the dimensional model of psychopathology (13), mental health presupposes not just the absence of mental illness, but also high degree of subjective well-being (12, 14). Clearly, the above mentioned adverse psychological and behavioural tendencies manifest as maladaptive, individually and socially harmful behaviours that affect the mental well-being of individual subjects, before impacting society at large. Thus, even if they are not in a categorical sense mental disorders, psychiatrists are still unwittingly faced with their individual and social consequences.

Second, as recent research has amply demonstrated, the cognitive and neural mechanisms that underly these phenomena—such as the intolerance of uncertainty, emotional dysregulation, cognitive biases, impulsivity, the dysregulation of valuation and decision-making—are the very same mechanisms that have been discovered to be causally implicated in the pathogenesis of a variety of mental disorders. They exist on a continuum with a range of mental disorders, as will be discussed below.

These points are perhaps best illustrated with a short vignette: Martin is a 40-year-old technician who recently lost his job and lives alone in a small town in an economically depressed area. His social relationships mainly take the form of interacting with online communities of like-minded individuals. He spends most of his time devouring alternative online media outlets, and contributing to online forums and blogs, which includes spreading fake-news and conspiracy theories. Although his personal experience with ethnic minorities is limited to contact with two gypsy families in his neighbourhood and the Vietnamese greengrocer in a shop that he frequents, he readily admits to feelings of suspicion and hatred towards Jews, Arabs, and—above all—refugees, who, he believes, are infiltrating the country with the aim of destroying it. Since the beginning of the COVID pandemic, he has steadfastly refused to follow public health measures, believing the pandemic is a hoax. Prone to constant feelings of anger and anxiety, his problems have worsened since he lost his job several months ago: he experiences bouts of anger, anxiety, and a feeling of helplessness almost every day. He alleviates these feelings with daily doses of cheap alcohol and recently also with benzodiazepines obtained from an online dealer. Having repeatedly posted online material celebrating the killing of Muslims and gypsies, he has been targeted by the police's cyber-crime unit and is currently facing criminal prosecution.

Whether his condition is best characterised as “problems in living” (15), as “harmful mental dysfunction” (16), or perhaps as “clinical psychological problems” (17), a condition that requires interventive treatment, is a moot point. He may never receive any psychiatric diagnosis, and a forensic psychiatric evaluation (should it come to that) would most likely rule out a mental disorder as the cause of his criminal behaviour. Yet, his is clearly a state of compromised mental health, involving both subjective mental suffering and personally disastrous as well as socially harmful behavioural choices, which affect other people and as a consequence increase the toxicity of the social environment. What is critical to Martin's condition is how his predicament is unfolding as he attempts to make sense of and cope both with his real personal lived experiences (such as his precarious existence) and with the reality of the outside world, as constructed by the various media to which he willingly exposes himself.

## Media, Uncertainty, and Stress

A major consequence of the above-mentioned global problems and the social construction of reality by the media is a massive increase in the uncertainty, unpredictability, and uncontrollability that characterises the world at large and, consequently, individual lives as well. It is well-known that the media is dominated by negative information and people display a negativity bias towards the news (18, 19). Across a number of studies, the media news have been consistently identified as a source of chronic stress and decreased mental well-being (20–22). This is even worse in the case of content disseminated by alternative outlets and weaponised artificial intelligence propaganda, whose very purpose is to increase people's uncertainty about the state of the world and each individual's prospects within it. This impacts mental well-being—and potentially psychopathology—principally in two interlinked ways.

First, it is well established that humans display sustained vigilance and defensive responding under conditions of uncertainty (23, 24). Recent evidence suggests that an intolerance of uncertainty is a critical transdiagnostic component of internalising psychopathology across a range of mental disorders (25, 26). Computations of subjective estimates of uncertainty predict acute stress response in humans (27) as well as depressive symptoms (28). While space constraints preclude more extensive discussion, it should be noted that the negative effects of uncertainty and unpredictability to a large extent depend on the individual subject's mental construction of the future. The key cognitive mechanisms include an episodic and semantic simulation of future events (29), whose links to psychopathology have recently been extensively examined (30, 31). Since imagining aversive events has emotionally negative consequences, internal simulations themselves incur some of the same costs as real-world experience (32). The aversive reaction is then the result of both the perceived threat to one's motivations and goals and to a decreasing ability to make meaningful sense of a changing and volatile social environment. People experiencing uncertainty and the aversive feelings that attend it will engage in actions to reduce it by initiating processes of compensatory control in an effort to imbue the world with order and predictability (33, 34).

Second, the uncontrollability and inescapability of both real and future imagined states of the world—and one's personal prospects in it—acts in predisposed individuals as a potent source of chronic stress. Indeed, in current theorising, stress itself is regarded as a form of uncertainty (35). If, following a recently proposed model, chronic stress is conceptualised as arising from a generalised perception of unsafety (36), the effect of the media can be seen as constructing the world as unsafe by default. Chronic stressors, characterised by uncontrollability and inescapability, have long been recognised as a major aetiological factor in depressed affect (37–39). This no longer pertains just to stressors at the proximal level of existence (such as health problems, interpersonal relations, financial difficulties and job insecurity etc.), but includes the distal and more abstract contextual level as well.

Furthermore, chronic stress has been found to disrupt neuroplasticity (40–42), a consequence of which is a decrease in psychological and cognitive flexibility. Psychological and cognitive inflexibility is a transdiagnostically relevant aethiological factor that is correlated and co-occurs with a number of cognitive and behavioural processes that underlie and maintain psychopathology (43, 44). Rigid cognitions are connected with a tendency towards negative appraisals of stressful situations (45, 46). Cognitive inflexibility predisposes for ruminative thought patterns in depression, anxiety disorders, and obsessive-compulsive disorder, and subjects with high cognitive inflexibility typically struggle to switch their attention away from internally-focused negative rumination (47, 48).

## Cognitive Inflexibility and Delusion-Like Beliefs

Cognitive inflexibility in itself, along with accompanying cognitive deficits, is a strong predisposing factor for the development of rigid beliefs and attitudes, which are what to varying degrees underlie almost all the problematic psychological and behavioural tendencies that were mentioned at the beginning of this article. Psychological models, such as the uncertainty-identity theory (49) and compensatory control theory (50, 51), have elaborated on how ideological inflexibility and extremism stems from a defensive need to alleviate uncertainty. Meanwhile, growing empirical evidence on the cognitive underpinnings of political ideologies confirms that cognitive rigidity is indeed linked to ideological extremism, partisanship, and dogmatism (52).

Furthermore, there has long been observed an association between belief inflexibility and delusions (53, 54). In some instances, rigidly held beliefs and attitudes acquire a delusional quality and can best be accounted for as instances of over-valued ideas (55, 56), or shared delusion-like beliefs (6, 7). In the realm of conspiratorial thinking, these rigid ideations may correspond to the clinical characteristics of an induced delusional disorder (or “*folie à deux*”)—a rare psychiatric condition in which an “inducer” (primary patient) transmits his or her delusional beliefs to another subject; both then share the same delusional ideation. As proposed by Dewhurst and Todd: (i) the persons involved should be closely associated, (ii) the content of the delusions should be identical or very similar, and (iii) the persons involved should accept, share, and support each other's delusions (57–59). The situation of the proximity of the inducer and followers and the simultaneous separation from other people who could offer an alternative, corrective point of view is now, because of the contemporary media echo chambers, even more extreme and beyond the scope imagined by the authors of the original definition.

Delusion-like beliefs are frequently found in the general population, spanning the continuum between mental illness and normalcy (60). While a variety of “tightened beliefs under uncertainty” (61), which extend into delusion-like beliefs, are in principle correctly positioned outside the sphere of psychiatric nosology, their underlying mechanisms at the systems and neural level provide a definite link to mental illness. One critical component of this link is what recent computational psychiatry call the “strong priors” model of hallucinations and delusions (62, 63). Tightened beliefs result in a limited number of rigid interpretation schemes [that is, priors at high levels of abstraction in a hierarchically structured environment (64)], which are typically observed among people with delusional and compulsive thoughts. Such high-level conceptual and belief priors about the world become strong and resistant to updating (65). As noted, people who are experiencing uncertainty will engage in actions to reduce it and the aversive feelings it generates by initiating processes of compensatory control. However, individuals cannot always resolve uncertainty by reconstructing their internal model of the world. A tightening of beliefs and ideas that is the result of a cognitive rigidity instantiated by strong priors can then be understood as an (ultimately maladaptive) defensive strategy for coping: to conserve energy and to avoid any further aversive emotional reactions brought about by the untolerable uncertainty. It can be seen as a shortcut in the process of active inference, the process of trying to make sense of an increasingly complex and uncontrollable state of affairs (the world) and one's own position in it.

## Cognitive Infexibility and Rigid Beliefs at the Society-Wide Level

A fundamental challenge for psychiatry is that aversive emotional reactions and a tightening of beliefs under the conditions of massive uncertainty and uncontrollability that media representations of social reality produce no longer concern just lonely violent extremists or fringe conspiratorial movements that subsist on fake news, as these reactions and beliefs are now being observed on a mass scale. This shift is being driven by the information infrastructure with rapid diffusion of media content that provide competing and incompatible constructions of social reality (66–68). As reactions to the COVID-19 pandemic have dramatically shown, it is increasingly being observed to also affect people who to now have not been drawn to fake news or inclined towards conspirational thinking or delusion-like beliefs, but who are nonetheless finding it difficult to adjust to and cope with the multiple burdens of global threats which impinge on their lives directly.

Importantly, a tightening of beliefs under conditions of chronic stress and uncertainty does not automatically translate into maladaptive or deviant social behaviour. Such behaviours, in any case, can take a range of forms, from the relatively innocuous (sharing fake news via e-mail) to the more consequential, such as refusing to adhere to public policy measures (wearing face masks during a pandemic) and offensive, deviant, and ultimately even violent acts. Cumulatively, such acts and behaviours threaten the stability of society as such, and by creating an increasingly toxic social environment they have downstream consequences for the clinical practise of psychiatry. However, for such behaviours to arise, further factors, such as the dysregulation of decision-making and cognitive control systems—e.g., the inhibition of habitual or impulsive responses, the inhibition of flexible updating and switching of behavioural dispositions, the dysfunctional emotional regulation and others (69–71)– must also be present. One of the topmost priorities for research is to identify the mechanisms and situational triggers through which rigid beliefs may turn to maladaptive and deviant behaviour.

## Facing the Challenge

How can psychiatric research and practise respond to this challenge? On a conceptual level, psychiatry needs to embrace (the not so new) position that mental states have a collective dimension (72, 73) and devote substantially more attention to the problem of how individual mental health is dynamically constrained and affected by interactions between individual minds and brains in a social space. It also requires psychiatric research to be increasingly interlinked with relevant research domains in the social sciences and in media and communication studies. From a public-health and policy perspective, the main issue is to build resilience against the adverse consequences of media-transmitted stressors. Here psychiatry should much more actively engage in efforts to mitigate the amplifying effects of the media in spreading stress and uncertainty and to address the downstream adverse mental and behavioural consequences of this. It needs to be more involved in areas such as media education and proactive policies targeting the spread of disinformation.

Even greater potential for action may arise at the level of fostering individual protective factors and resilience against media-induced adversity. Given the identified mechanisms contributing to the development of maladaptive responses, these preventive and well-being supporting strategies should be primarily based on the promotion of psychological and structural and functional neural plasticity, which could help to acquire and foster neural resilience in people and could thereby have a beneficial effect on socioemotional well-being (74, 75). Remediation strategies should be aimed at the relaxation of pathologically over-weighted “priors” or habits of mind and behaviour (76).

It would be naïve to expect that global environmental and socio-political stressors will have a less stressful impact on communities and individuals in the decades to come. The adverse psychological and behavioural tendencies discussed above that have arisen largely in response to these stressors are thus unlikely to recede. Unless psychiatric research and practise accept the major role they need to play in responding to these negative phenomena, clinicians will increasingly be overwhelmed by their mental health sequelae.

## Data Availability Statement

The original contributions presented in the study are included in the article/supplementary material, further inquiries can be directed to the corresponding author/s.

## Author Contributions

LK drafted the article. JH contributed to writing. All authors contributed to the article and approved the submitted version.

## Funding

This work was supported by the Czech Science Foundation (project no. 20-13458S).

## Conflict of Interest

The authors declare that the research was conducted in the absence of any commercial or financial relationships that could be construed as a potential conflict of interest.

## Publisher's Note

All claims expressed in this article are solely those of the authors and do not necessarily represent those of their affiliated organizations, or those of the publisher, the editors and the reviewers. Any product that may be evaluated in this article, or claim that may be made by its manufacturer, is not guaranteed or endorsed by the publisher.
